# Supplementary material for: Cathepsins Trigger Cell Death and Regulate Radioresistance in Glioblastoma
Source: Cells. 2022 Dec 17;11(24):4108. doi: 10.3390/cells11244108 (PMC9777369; doi:10.3390/cells11244108)
Supplement: Supplementary file 1 [file cells-11-04108-s001.zip › supplementary.pdf]

Figure S1

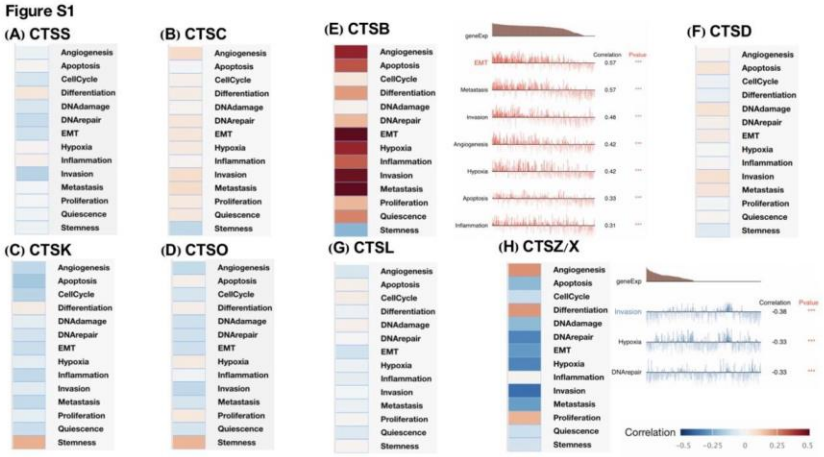


**Supplementary Figure1.** The relationship between cathepsins and different functional states of GBM was explored based on the CancerSEA database . *p < 0.05; **p < 0.01; ***p < 0.001. Bar graphs show the number of datasets where cathepsins are significantly associated with the corresponding state. Red indicates positive correlation, blue indicates negative correlation.

Figure S2

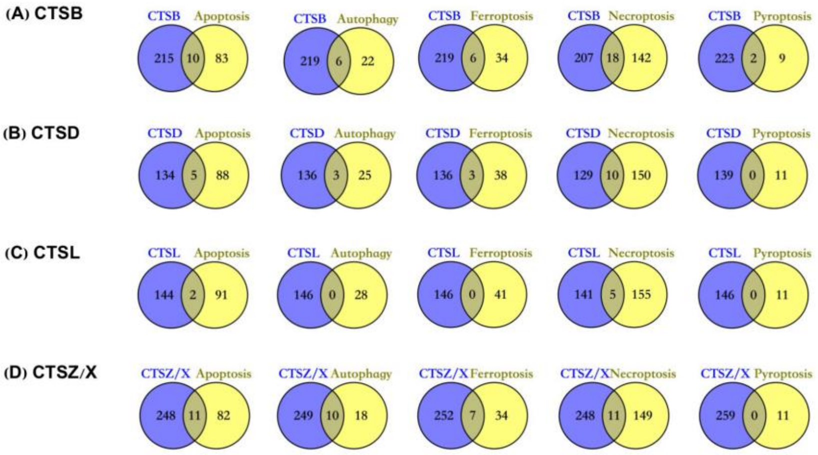


**Supplementary Figure2.** Intersection of interacting proteins of CTSB, CTSD, CTSL and CTSZ/X with cell death-related genes.
